# Towards a taxonomy of behavior change techniques for promoting shared decision making

**DOI:** 10.1186/s13012-020-01015-w

**Published:** 2020-08-20

**Authors:** Titilayo Tatiana Agbadjé, Hélène Elidor, Milena Sia Perin, Rhéda Adekpedjou, France Légaré

**Affiliations:** 1 Canada Research Chair in Shared Decision Making and Knowledge Translation, Université Laval, Quebec, Canada; 2 Centre de recherche en santé durable (VITAM), Quebec, QC Canada; 3 Centre Intégré Universitaire de Santé et Services Sociaux de la Capitale-Nationale (CIUSSS-CN), Quebec, QC Canada; 4 Faculty of Nursing, University of Campinas, Sao Paulo, Brazil; 5 Department of Family Medicine and Emergency Medicine, Faculty of Medicine, Université Laval, Quebec, Canada

**Keywords:** Behavior change techniques, Functions, Shared decision making, Implementation interventions, Taxonomy, Behaviour change wheel

## Abstract

**Background:**

There is little information about the functions and behavior change techniques (BCTs) needed to implement shared decision making (SDM) in clinical practice. To guide future implementation initiatives, we sought to develop a BCT taxonomy for SDM implementation interventions.

**Methods:**

This study is a secondary analysis of a 2018 Cochrane review on interventions for increasing the use of shared decision making by healthcare professionals. We examined all 87 studies included in the review. We extracted relevant information on each study intervention into a spreadsheet. Coders had undergone a training workshop on intervention functions and online training on BCT Taxonomy version 1 (BCTTv1). We performed functions and BCTs coding trials, and identified coding rules. We used Michie’s guide for designing behavior change interventions to code the functions and BCTs used in the interventions. Coders met to compare coding and discrepancies were discussed until consensus was reached. Data was analyzed using simple descriptive statistics.

**Results:**

Overall, 7 functions, 24 combinations of functions and 32 BCTs were used in the 87 SDM implementation interventions. The mean of functions per intervention was 2.5 and the mean of BCTs per intervention was 3.7. The functions Coercion and Restriction were not found. The most common function was Education (73 studies). Three combinations of functions were most common (e.g: Education + Persuasion, used in 10 studies). The functions associated with more effective SDM implementation interventions were Modeling and Training. The most effective combination of functions was Education + Training + Modeling + Enablement. The most commonly used BCT was Instruction on how to perform the behavior (43 studies). BCTs associated with more effective SDM implementation interventions were: Instruction on how to perform the behavior, Demonstration of the behavior, Feedback on behavior, Pharmacological support, Material reward, and Biofeedback. Twenty-five BCTs were associated with less effective SDM implementation interventions. Four new BCTs were identified: General information to support the behavior, Tailoring, Exercises to conceptually prepare for the behavior, and Experience sharing and learning.

**Conclusions:**

We established a BCT taxonomy specific to the field of SDM to guide future SDM implementation interventions. Four new BCTs should be added to BCTTv1.

Contributions to the literature

- While a taxonomy of behavior change techniques (BCTs) has been developed to report behavior change interventions, there is currently no information on BCTs or strategies that have been or should be used for shared decision making (SDM) implementation interventions.
- From this study, we now know the overall approaches (functions, combinations of functions, and BCTs) that have been most effective in past efforts to increase shared decision making among patients and health professionals.
- We developed a list of BCTs useful specifically in strategies for increasing SDM.
- These findings will inform future SDM intervention implementation initiatives for better results.

## Background

In the area of healthcare, when there are multiple treatments or screening options, best practice requires health professionals (HPs) to involve patients in shared decision making (SDM) [1–3]. SDM has been described as an interpersonal and interdependent process whereby HPs support clients in making decisions that are informed by best evidence and by what matters most to them. They thus collaborate to make decisions about the patient’s health [1, 2]. Evidence suggests that SDM improves the healthcare experiences of both clients [4] and HPs [5] and leads to better healthcare processes [6], client outcomes [4], and lower health costs [7]. SDM may also contribute to reducing the overuse of ineffective care options and increasing uptake of effective ones [8]. It could play a crucial role in reducing harms and increasing client safety [4] and seems to promote informed consent [9], which is fundamental to client/family-centered care and is considered an ethical imperative [10]. Moreover, decision aids (also known as SDM tools) can help patients become better informed about their healthcare options and have more realistic expectations and clarity about their values [8] However, despite the many advantages associated with SDM, it has not yet been widely adopted in practice [11].

A variety of implementation strategies have been attempted to change the behavior of HPs to ensure they deliver optimal care to patients. These include the distribution of printed educational materials, educational meetings, audits and feedback, reminders, educational visits, and patient-mediated interventions [12]. A 2018 Cochrane Systematic Review identified 87 SDM implementation trials and found that overall, implementation interventions that targeted both HPs and patients were more promising than those that only targeted HPs or patients [11]. However, this review did not attempt to classify or explore the detailed components of the diverse implementation interventions nor to inform readers about their theoretical underpinnings, thus reducing the potential impact of its results on clinical care.

Intervention development science identifies various intervention functions and behavior change techniques (BCTs) that can be used to change clinical behavior [13, 14]. Interventions can have 9 possible “functions,” defined as “a broad category of means by which an intervention can change behavior” [13], while a BCT is defined as “an active component of an intervention designed to change behavior” [13, 14]. In 2013, Michie and colleagues proposed an international taxonomy of 93 hierarchically clustered BCTs [15]. This evidence-based taxonomy was developed with the aim of building an international consensus for reporting behavior change interventions and to support the creation of theory-informed implementation interventions [15]. We therefore hypothesized that all SDM implementation interventions could be mapped on specific functions and BCTs [13–15]. While there are some BCT taxonomies specific to certain clinical areas, such as smoking cessation [16] and alcohol consumption reduction [17], there is currently no information on what functions and BCTs have been used in SDM implementation interventions or standard guidance on which ones should be used. Therefore, to guide future SDM implementation initiatives, we sought to develop a BCT taxonomy specific to SDM implementation interventions. Specific objectives were 1) to determine the functions of interventions reported in studies identified in a 2018 Cochrane review that aimed to increase the use of shared decision making by HPs, 2) to determine which combinations of functions were most commonly used in SDM implementation interventions, 3) to determine the BCTs in interventions reported in these studies, and 4) to determine which functions, combinations of functions, and BCTs of interventions reported in these studies were associated with a positive effect.

## Methods

### Study design

This study is a secondary analysis of a 2018 Cochrane review entitled “Interventions for increasing the use of shared decision making by healthcare professionals” [11]. In the present study, we examined all 87 studies included in the review. The studies explored SDM implementation interventions in many contexts. Details related to information sources, searches, study selection, data collection process, data items, risk of bias in individual studies, summary measures, risk of bias across studies, and synthesis of results (including meta-analysis results) of the primary source of data are available in the full text of the review [11].

The study population of the Cochrane review was any healthcare professional, including professionals in training, and patients, including healthcare consumers and simulated patients. The interventions were organized into 3 target categories: interventions targeting patients, interventions targeting healthcare professionals, and interventions targeting both patients and healthcare professionals. The principal effect sought, or primary outcome, was the use of SDM, measured using objective observer-based outcome measures (OBOMs) or patient-reported outcome measures (PROMs). Secondary outcomes were affective-cognitive outcomes (e.g., decisional conflict), behavioral outcomes (e.g., adherence to a decision made), health outcomes (e.g., depression), and process outcomes (e.g., consultation length) [11].

There are currently no reporting guidelines for secondary analyses of systematic reviews. Thus, we used the Preferred Reporting Items for Systematic Reviews and Meta-Analyses (PRISMA) checklist [18]. Also, we were inspired by other published articles on various existing taxonomies. See Additional file 1 for the PRISMA checklist.

### Data collection

#### Extraction

For each study, we extracted the following information into a spreadsheet: name of the first author, year, target population (e.g., patients, providers, or both). We also extracted target behavior, type of intervention, and content of the intervention. The extraction was performed by one author (TA) and verified by two more authors (HE, MSP). They reached consensus about discrepancies, and any disagreement was resolved through discussion among all authors. They then coded functions into this initial extraction spreadsheet.

#### Coding procedures

***Theoretical underpinnings*** Thirty-one out of the 87 studies included in the Cochrane review used or referred to a conceptual framework (e.g., Ottawa Decision Support Framework) [11]. The present study was based on the Behavior Change Wheel (BCW), an 8-step guide for designing and evaluating interventions that synthesize 19 behavior change frameworks [13, 14]. The BCW is based on the COM-B behavior model, which proposes that capacity (C), opportunity (O), and motivation (M) are the 3 conditions essential to performing a behavior (B) [13, 14]. According to the BCW, changing a behavior involves using one or more of 9 “intervention functions” to address deficiencies in one or more of these 3 conditions [13, 14]. Intervention functions are Education (knowledge-based), Training (skill-based), Environmental restructuring, Modeling, Persuasion, Coercion, Incentivization, Restriction, and Enablement [13, 14]. Processes that regulate behavior may also be changed using one or more BCTs. BCTs are components of an intervention that are observable, replicable, and irreducible [13, 14], and a taxonomy of 93 BCTs found in behavior change interventions has been proposed [13–15]. In this secondary analysis, we were inspired by elements of the guide for designing behavior change interventions by Michie and colleagues (2011, 2014) to determine the functions and BCTs used in SDM implementation interventions.

***Coding of functions*** We used step 5 of Michie’s guide (definition of intervention functions), as a reference for the identification and coding of intervention functions [13, 14]. Two coders (TA, HE) individually identified and coded interventions, and a third coder (MSP) validated their coding. The coders were researchers experienced in SDM, implementation science, and intervention design who had attended a training workshop on the intervention functions based on Michie’s book on the Behavior Change Wheel [14] and on literature on the various intervention approaches [19–29]. Each coder individually analyzed each article to identify which of the 9 possible functions were used in the intervention. For example, in interventions attempting to increase SDM skills among healthcare professionals, Training was often the function identified. Other interventions focused on Restructuring the environment to make SDM possible. After coding, 11 meetings were held until consensus was achieved on the functions identified.

***Coding of BCTs*** Similarly, the identification and coding of BCTs were based on the taxonomy of 93 BCTs in the guide on designing behavior change interventions (step 7, definition of BCTs) [13, 14]. The coding was performed individually by 3 coders (TA, HE, MSP). Each coder had separately undergone the BCT Taxonomy version 1 (BCTTv1) Online Training [30]. After training, they separately performed BCT coding trials on 7 studies and compared their coding. Following this, they came to an agreement on how to code difficult portions of the study texts [13]. For example, “coaching session” was coded as Practical social support. For both functions and BCTs, authors adhered to the following rules [31]: 1) code the content of the intervention but not the aim, 2) do not code sentences unrelated to the target behavior, 3) consider that the target behavior may encompass several behaviors, and 4) list sentences whose coding is problematic and any difficulties in using the v1 taxonomy for coding. Pairs of coders then discussed the results of their function and BCT coding for each article (TA and MSP, or TA and HE) until consensus was reached. Any discrepancies in coding between pairs were resolved by a third coder. If a technique was found that did not match any of the 93 BCTs in the guide, we proposed a new BCT. BCT codes were entered into a Word file and after consensus, moved into the initial spreadsheet for analysis.

***Data analysis*** In the context of SDM, 3 overarching categories of implementation intervention can be identified: 1) those targeting patients, 2) those targeting healthcare professionals, and 3) those targeting both. We therefore performed subgroup analyses based on the population targeted. We extracted descriptive statistics (mean, median, frequencies, and proportions) on all identified functions and BCTs used in SDM implementation interventions. We computed the most frequent functions and BCTs used as well as combinations of functions found within each category (target population). We also identified which function, combination of functions and BCTs mapped on to SDM implementation interventions that had been identified as either effective or ineffective when implementing SDM in clinical practice. Given the heterogeneity of outcome measures and the objective of this secondary analysis, we performed only a descriptive summary of data. Analyses were performed on SAS software version 9.4.

## Results

### Characteristics of studies

Briefly, of the 87 studies evaluated in the Cochrane review, 15 (17.2%) of them targeted only healthcare professionals, 44 (50.6%) targeted only patients, and 28 (32.2%) targeted both populations [11]. Many interventions used combinations of functions (in 69 studies) and combinations of BCTs (in 73 studies) rather than single ones.

### Functions used in SDM implementation interventions

Overall, the SDM implementation interventions included in the 87 studies mapped on to 7 out of the 9 functions identified by Michie et al. (2011, 2014) [13, 14]. The functions Coercion and Restriction were absent. Each implementation intervention had between 1 and 5 functions. Most interventions (28 studies) had 2 functions. The mean number of functions per intervention was 2.5. The 3 functions most frequently found were Education (73 studies, 84%), Enablement (49 studies, 56%), and Training (40 studies, 45%) (Table 1). In the context of SDM, an example of Persuasion was informing the patient of their absolute risk for stroke and myocardial infarction compared with the overall age- and sex-adjusted population risk. An example of Enablement was participants practicing engaging in conversations with residents about preferences in routine care situations.

**Table 1:** Functions and effectiveness of shared decision making implementation interventions according to the target population

Functions	Effect	Total	Population target (***N*** = 87)		
		*N* = 87 (%)	HP *N* = 15 (%)	Patients *N* = 44 (%)	Both *N* = 28 (%)
**Education**		73 (84)	8 (53)	41 (93)	24 (86)
	Positive	32 (37)	4 (26.5)	12 (27)	16 (57)
	No effect	40 (46)	4 (26.5)	29 (66)	7 (25)
	Missing data	1 (1)	0 (0)	0 (0)	1 (4)
**Enablement**		49 (56)	12 (80)	19 (43)	18 (64)
	Positive	21 (24)	7 (47)	3 (7)	11 (39)
	No effect	27 (31)	5 (33)	16 (36)	6 (21)
	Missing data	1 (1)	0 (0)	0 (0)	1 (4)
**Training**		40 (46)	14 (93)	5 (11)	21 (75)
	Positive	21 (24)	6 (40)	0 (0)	15 (53)
	No effect	18 (21)	8 (53)	5 (11)	5 (18)
	Missing data	1 (1)	0 (0)	0 (0)	1 (4)
**Modeling**		17 (20)	6 (40)	0 (0)	11 (39)
	Positive	12 (14)	3 (20)	0 (0)	9 (32)
	No effect	5 (6)	3 (20)	0 (0)	2 (7)
**Persuasion**		13 (15)	0 (0)	12 (27)	1 (4)
	Positive	6 (7)	0 (0)	5 (11)	1 (4)
	No effect	7 (8)	0 (0)	7 (16)	0 (0)
**Environmental restructuring**		10 (12)	4 (27)	1 (2)	5 (18)
	Positive	4 (5)	1 (7)	1 (2)	2 (7)
	No effect	6 (7)	3 (20)	0 (0)	3 (11)
**Incentivization**		9 (10.3)	0 (0)	3 (7)	6 (21)
	Positive	4 (4.6)	0 (0)	1 (2)	3 (10.5)
	No effect	5 (5.7)	0 (0)	2 (5)	3 (10.5)
**Coercion**		0 (0)	0 (0)	0 (0)	0 (0)
**Restriction**		0 (0)	0 (0)	0 (0)	0 (0)

*HP* health professional, *N* number of studies

Among the 15 studies in which interventions targeted HPs only, the function Training was present in 14 studies and was the most common function (93.3%), either alone or in combination. Among the 44 studies in which interventions targeted patients only, the function Education was present in 41 studies and was the most common function (93.2%), either alone or in combination. Among the 28 studies in which interventions targeted both patients and HPs, the 2 most common functions, alone or in combination, were Education (24 studies, 85.7%) followed by Training (21 studies, 75%) (Table 1). Also, see Additional file 2 for examples of each function used in the SDM implementation interventions.

### Combinations of functions used in SDM implementation interventions

In 69 studies (79% of the total), interventions focused on more than one function. We found 24 combinations of functions. The 3 most frequent combinations were: 1) Training + Enablement (10 studies, 11.5%) and 2) Education + Persuasion (10 studies, 11.5%), and 3) Education + Training + Modeling + Enablement (10 studies, 11.5%) (Table 2).

**Table 2:** Combinations of functions used in shared decision making implementation interventions and their frequency

Combinations of functions						n_Comb	***N***	Effect	
1.	Training	Environmental restructuring	–	–	–	2	1	Positive	0
								No effect	1
2.	Education	Enablement	–	–	–	2	10	Positive	1
								No effect	9
3.	Education	Incentivization	–	–	–	2	2	Positive	0
								No effect	2
*4.	Education	Training	–	–	–	2	2	Positive	2
								No effect	0
5.	Training	Enablement	–	–	–	2	2	Positive	1
								No effect	1
6.	Education	Persuasion	–	–	–	2	10	Positive	5
								No effect	5
*7.	Education	Modeling	–	–	–	2	1	Positive	1
								No effect	0
8.	Training	Environmental restructuring	Enablement	–	–	3	2	Positive	1
								No effect	1
9.	Education	Environmental restructuring	Enablement	–	–	3	2	Positive	1
								No effect	1
10.	Education	Training	Environmental restructuring	–	–	3	1	Positive	0
								No effect	1
11.	Training	Incentivization	Enablement	–	–	3	1	Positive	0
								No effect	1
12.	Education	Training	Enablement	–	–	3	9	Positive	3
								No effect	5
								Error	1
13.	Education	Incentivization	Enablement	–	–	3	2	Positive	1
								No effect	1
*14.	Education	Training	Incentivization	–	–	3	1	Positive	1
								No effect	0
*15.	Training	Modeling	Incentivization	–	–	3	1	Positive	1
								No effect	0
*16.	Education	Training	Modeling	–	–	3	1	Positive	1
								No effect	0
17.	Training	Modeling	Enablement	–	–	3	2	Positive	1
								No effect	1
18.	Education	Persuasion	Enablement	–	–	3	3	Positive	1
								No effect	2
19.	Education	Modeling	Enablement	–	–	3	1	Positive	0
								No effect	1
20.	Education	Training	Environmental restructuring	Enablement	–	4	2	Positive	1
								No effect	1
21.	Education	Training	Modeling	Environmental restructuring	–	4	1	Positive	0
								No effect	1
22.	Education	Training	Incentivization	Enablement	–	4	1	Positive	0
								No effect	1
*23.	Education	Training	Modeling	Enablement	–	4	10	Positive	8
								No effect	2
*24.	Education	Training	Environmental restructuring	Incentivization	Enablement	5	1	Positive	1
								No effect	0

*n_Comb* number of functions in the combination

*Indicates that the combination is more associated with studies showing a positive effect than with no-effect studies

### Behavior change techniques used in SDM implementation interventions

**Fig. 1:**
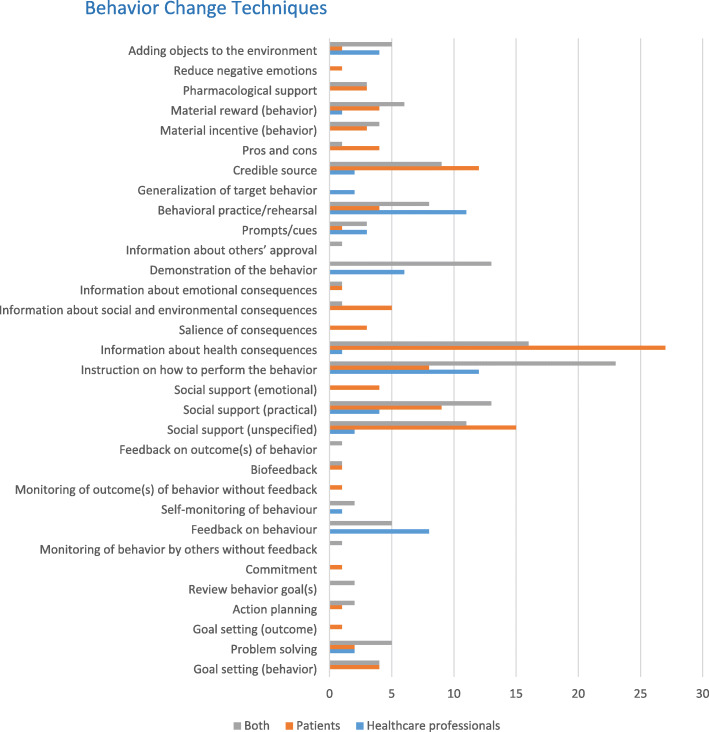
Frequency of behavior change techniques used in shared decision making implementation interventions by the target population

Up to 10 BCTs were used in each single SDM implementation intervention. Overall, 32 BCTs were used out of the 93 BCTs in the BCTTv1 (see Fig. 1 and Additional file 3—BCTs used in each SDM implementation intervention). Most interventions (21 studies) used 4 BCTs. The mean number of BCTs per intervention was 3.7. The 10 BCTs most commonly used, either alone or in combination, were Instruction on how to perform the behavior (53 studies, 41.4%), Information about health consequences (46 studies, 35.9%), Social support (unspecified) (29 studies, 22.6%), Social support (practical) (27 studies, 21.1%), Credible source (24 studies, 18.7%), Demonstration of the behavior (23 studies, 18%), Behavioral practice/rehearsal (22 studies, 17.2%), Feedback on behavior (15 studies, 11.7%), Adding objects to the environment (13 studies, 10.2%), and Problem-solving (9 studies, 7%) (see Additional file 4).

In the context of SDM, an example of Feedback on behavior was researchers giving confidential written feedback on audio-recorded participant input in a workshop. An example of Adding objects to the environment was giving participants an audiotape and asking them to record a consultation.

Studies used a wide variety of BCT combinations. Of the 73 studies that used more than one BCT, we did not obtain high enough “n” values of the same combinations to draw conclusions. In interventions targeting HPs, we identified 14 BCTs, and the most common was Instruction on how to perform the behavior. In interventions targeting patients, we identified 25 BCTs and the most common was Information about health consequences. In interventions targeting both patients and HPs, we identified 25 BCTs and the most common was Instruction on how to perform the behavior. Nine BCTs were common to all 3 populations. See Additional file 2 for examples of each BCT used in the SDM implementation interventions.

### New behavior change techniques found in SDM implementation interventions

In one of the SDM implementation studies, we were unable to code the BCTs. In another, we were unable to identify the function, while in a third, we were unable to identify either the functions or the BCTs. Also, in more than half of the studies there were portions of sentences we were not able to code. Some of the BCTs or strategies used in SDM interventions did not fit the definitions in the BCTTv1. We analyzed these strategies to create and define 4 new BCTs: General information to support the behavior (e.g., information about diagnosis and treatment), Tailoring (e.g., integrate personal risk information or personal characteristics), Exercises to conceptually prepare for the behavior (e.g., knowledge quizzes, values clarification exercises), and Experience sharing and learning (e.g., discuss experiences of health decisions or training with others) (Tables 3 and 4).

**Table 3:** Definition of four new behavior change techniques with examples

New BCT name	Proposed definition	Example	Quote	Frequency of use ***N*** (%)*	Closest existing BCT(s) in BCTTv1	How the new BCT differs from the closest existing BCT
Providing background information	Provide general information, e.g., about diagnosis and treatment options, to facilitate the behavior.	Explain in a cardiovascular diseases (CVD) health booklet details about the diagnosis, symptoms of the disease, and describe treatment options.	“The booklet provides general information on CVD, CVD-risk factors and lifestyle changes, and medication options for improving cardiovascular health.” Lalonde, 2006	43 (49.4%)	BCT: Information about health consequences Definition: Provide information (e.g., written, verbal, visual) about health consequences of performing the behavior. Group: Natural consequences	Providing background information about the problem requiring a behavior change is not about the different types of consequences of the behavior, but is about other relevant background information such as the cause, symptoms, or prognosis of a disease.
Tailoring	Provide personalized information or a vehicle (e.g., brochure, video, app) adapted to the individual or specific group’s needs (e.g., personal risk information)	Provide a personal risk assessment of the patient’s chance of fracture; adapt video clips to a patient’s individual response patterns.	“The Osteoporosis Choice decision aid provides the patient’s individualized 10-year risk estimate risk of having a major osteoporotic fracture (i.e., clinical [“symptomatic”] spine, forearm, hip, or shoulder fracture).” Montori, 2011 “A scoring algorithm was developed to link questionnaire responses to specific skills so that the video clips presented to a viewer could be tailored to their individual response patterns and preference for a particular character.” Roter, 2012	27 (31.03%)	BCT: Problem solving Definition: Analyze, or prompt the person to analyze, factors influencing the behavior and generate or select strategies that include overcoming barriers and/or increasing facilitators (includes “Relapse Prevention” and “Coping Planning”). Group: Goals and planning	While “Problem solving” is conditional on problems that could arise when attempting to adopt the behavior, and depends on the individual to implement it, “Tailoring” is a mechanism built into an intervention that adapts it to each individual profile.
Mental preparation for the behavior	Provide conceptual exercises (includes quizzes and values clarification exercises) to better prepare for the behavior.	Ask someone to identify, clarify and prioritize their values and preferences regarding surgery.	“An exercise on values clarification in which patients ranked their personal goals associated with two major surgery types (autologous vs. implant) from 1 (does not reflect my personal values at all) to 5 (reflects my personal values very well).” Causarano, 2015	12 (13.8%)	BCT: Behavioral practice/rehearsal Definition: Prompt practice or rehearsal of the performance of the behavior one or more times in a context or at a time when the performance may not be necessary, in order to increase habit and skill. Group: Repetition and substitution	While “Behavioral practice/rehearsal” focuses on the target behavior and aims at increasing habit or skills, “Mental preparation for the behavior” targets sub-behaviors necessary at a more conceptual level and aims at preparing for a (future) behavior by reflecting on one’s personal life choices regarding the behavior.
Experience sharing and learning	Benefit from others’ experiences through testimony or share experiences with others about one’s own learning regarding the behavior.	Ask a woman who has already undergone chemotherapy treatment for breast cancer to share her experiences with a group of women who have been diagnosed with breast cancer to encourage them to follow the treatment.	“The modules include … vignettes from a racially diverse group of patients describing their experience with a particular test.” Schroy, 2011	7 (8.04%)	BCT: Information about others’ approval Definition: Provide information about what other people think about the behavior. The information clarifies whether others will like, approve or disapprove of what the person is doing or will do. Group: Comparison of behavior group	“Experience sharing and learning” goes further than approval or disapproval. It focuses on learning by sharing the experience of adopting the behavior. From the point of view of experience sharing, it aims to help the person contextualize, relativize and better deepen his or her own reflection in relation to someone else’s experience. From the point of view of learning it aims at consolidating learning.

*Over 87 studies,* BCT* Behavior change technique, *BCTTv1* Behavior change technique Taxonomy version 1, *N *number of studies.

**Table 4:** Taxonomy of behavior change techniques used in shared decision making implementation interventions

Grouping and BCTs	Status		
	***Found in effective studies***	***Found in ineffective studies***	***New***
**1. Goals and planning**			
Goal setting (behavior)		✓	
Problem solving	✓	✓	
Goal setting (outcome)	✓	✓	
Action planning	✓	✓	
Review behavior goal(s)		✓	
Commitment		✓	
Tailoring			✓
**2. Feedback and monitoring**			
Monitoring of behavior by others without feedback		✓	
Feedback on behavior	✓	✓	
Self-monitoring of behavior	✓	✓	
Monitoring of outcome(s) of behavior without feedback		✓	
Biofeedback	✓		
Feedback on outcome(s) of behavior		✓	
**3. Social support**			
Social support (unspecified)	✓	✓	
Social support (practical)	✓	✓	
Social support (emotional)	✓	✓	
**4. Shaping Knowledge**			
Instruction on how to perform the behavior	✓	✓	
Providing background information			✓
**5. Natural consequences**			
Information about health consequences	✓	✓	
Salience of consequences		✓	
Information about social and environmental consequences	✓	✓	
Information about emotional consequences		✓	
**6. Comparison of behavior**			
Demonstration of the behavior	✓	✓	
Information about others’ approval		✓	
Experience sharing and learning			✓
**7. Associations**			
Prompts/cues	✓	✓	
**8. Repetition and substitution**			
Behavioral practice/rehearsal	✓	✓	
Generalization of target behavior		✓	
Mental preparation for the behavior			✓
**9. Comparison of outcomes**			
Credible source	✓	✓	
Pros and cons	✓	✓	
**10 Reward and threat**			
Material incentive (behavior)	✓	✓	
Material reward (behavior)	✓	✓	
**11. Regulation**			
Pharmacological support	✓	✓	
Reduce negative emotions		✓	
**12. Antecedents**			
Adding objects to the environment	✓	✓	

*BCT* Behavior change technique

### Comparative statistics on functions and combinations of functions mapped on to the effectiveness of the SDM implementation interventions

In terms of the effectiveness of the interventions overall, 37 (43%) studies in the Cochrane review showed a positive effect in favor of the intervention group, 49 (56%) studies showed no effect in favor of the intervention group, and it was not possible to specify the effects of one study (1%) because of a unit of analysis error.

#### Functions in positive effect studies vs. in no effect studies (*n* = 87)

Each function was found both in studies showing a positive effect and those showing no effect. However, comparing the effects of studies by function, the two functions found in more studies showing a positive effect (compared to studies showing no effect) were Modeling (12 out of 17 studies focusing on modeling) and Training (21 out of the 40 studies focusing on training). The 2 functions found in more no effect studies (compared to positive effect studies) were Education (40 out of 73 studies focusing on Education) and Enablement (27 out of the 49 studies focusing on Enablement) (Table 1).

Comparing the effects of studies by function and by the different target populations, functions found in more studies showing a positive effect (compared to no effect) were in interventions targeting both patients and HPs: Education studies (16 studies showing positive effect vs. 7 studies with no effect), Enablement studies (11 studies vs. 6 studies), Training studies (15 studies vs. 5 studies), Modeling studies (9 studies vs. 2 studies), and Persuasion studies (1 study vs. 0 studies). However, Enablement studies also showed more positive effects (7 studies vs. 5 studies) in interventions targeting HPs as well as in interventions targeting both. Whatever the functions found, interventions targeting HPs only or patients only for the most part had more studies showing no effect than showing a positive effect (Table 1).

#### Combinations of functions in positive effect studies vs. in no effect studies (*n* = 87)

Out of 24 combinations, 8 combinations were found in more studies showing a positive effect (compared to studies showing no effect) and 16 were found in more studies showing no effect (compared to studies showing positive effect). The most effective combination was Education + Training + Modeling + Enablement (8 studies with positive effect vs. 2 studies with no effect). The least effective combination was Education + Enablement (9 studies with no effect vs. 1 study with positive effect) (Table 2).

### Comparative statistics on BCTs mapped on to the effectiveness of the SDM implementation interventions (*n* = 87)

Comparing the effects of studies by BCT, 5 BCTs were found in more studies showing a positive effect (compared to studies showing no effect), and 25 BCTs were found in more studies showing no effect (compared to those showing positive effects) and 2 BCTs were found in studies that showed as much positive effect as no effect.

The 5 BCTs either alone or in combination found in more studies showing a positive effect were Instruction on how to perform the behavior (22 studies showing a positive effect vs. 20 studies showing no effect), Demonstration of the behavior (12 studies vs. 6 studies), Feedback on behavior (8 studies vs. 5 studies), Pharmacological support (5 studies vs. 1 study), and Biofeedback (1 study vs. 0 studies) (see Additional file 4).

Of the 25 BCTs either alone or in combination found in more studies showing no effect (compared to positive effect), the 5 most frequent BCTs were Information about health consequences (23 studies with no effect vs. 21 studies with a positive effect), Social support (unspecified) (19 studies vs. 8 studies), Credible source (13 studies vs. 9 studies), Social support (practical), (13 studies vs. 12 studies), and Material reward (behavior) (7 studies vs. 3 studies) (see Additional file 4).

## Discussion

This secondary analysis aimed to propose a taxonomy of BCTs specific to SDM implementation interventions based on existing SDM implementation studies. We identified 7 functions, 24 combinations of functions (ranging from 2 to 5), and 32 BCTs. Two functions (modeling and training), 8 combinations of functions (e.g., Education + Training + Modeling + Enablement), and 5 BCTs (e.g., Instruction on how to perform the behavior) were most associated with interventions showing positive effects. Two functions (Education and Enablement), 16 combinations of functions (e.g., Education + Enablement), and 25 BCTs (e.g., Information about health consequences) were most associated with no effect interventions. The functions Coercion and Restriction were not found. We created 4 new BCTs.

First, the results of our study showed that 7 behavior change functions were used in SDM implementation interventions. Some of these behavior change functions seem better matched to the SDM implementation context than others. Our study not only identified the most effective functions, but also the most effective and ineffective combinations of functions in SDM interventions. While Michie et al. (2011, 2014) advise applying the APEASE criteria (affordability, practicability, effectiveness, acceptability, side effects and equity) when selecting an intervention strategy [13, 14], our findings suggest that researchers must also reflect on which combinations of functions are most relevant to their own specific context. Just as each intervention requires a specific combination of BCTs [32], it also requires a specific combination of functions to give the expected results.

Second, we noticed that 2 functions (Restriction and Coercion) were not used in SDM implementation interventions. This could be explained by the fact that Restriction and Coercion are not ethically compatible with the concept of SDM, which is in principle based on the willing cooperation of all parties [33]. This could change, however, with the increasing number of countries that have enacted laws or policies obliging physicians to adopt SDM [34]. For the function Environmental restructuring, the only BCT we found was the Addition of an object to the environment (e.g., putting a pile of decision aids on the doctor’s desk). Researchers could also experiment with other BCTs associated with this function, such as Restructuring the social environment with, for instance, interventions that encourage the view of SDM as the norm in medical consultations [35].

Third, careful analysis of the effectiveness of interventions by function and by target population in our study showed that SDM implementation interventions targeting both patients and healthcare professionals gave better results than those targeting just one population or the other. We found functions showing more positive effect than no effect (5 functions out of 7) in the interventions targeting both patients and healthcare professionals, while this trend was not present in interventions targeting health professionals only (where we found as many effective and ineffective interventions for most functions) or in interventions targeting patients only (where we found more ineffective interventions for most functions). Despite the presence of empty cells, the finding was the same when analyzing the effectiveness of interventions by BCT and by the target population. This clearly indicates that by targeting both the patient and the health professional, results are better than when the intervention is given just to one or the other. This is in line with the conclusions of the Cochrane review [11]. This also aligns well with the ecological approach to behavior change, which suggests that the more dimensions (in our case, targeted populations) that are considered in the development of the intervention, the more effective it will be [36]. Thus, researchers should consider all parties involved in the shared decision-making process to maximize the impact of their intervention.

Fourth, we created new BCTs for techniques we were not able to code using the current taxonomy. SDM is different in nature from other behaviors and contexts explored in the development of the current cross-behavior BCT taxonomy [15]. The desired behavior is most of the time *to make a decision* for one management option or another, rather than to adopt a behavior related to the management itself of the condition. To complete BCTTv1, we therefore identified and defined additional BCTs potentially relevant to the domain of SDM or other domains in which interventions make use of similar strategies. We believe that these additions to the taxonomy will be useful for SDM intervention designers, researchers, practitioners, authors of systematic reviews, and all those wishing to communicate or evaluate the content of SDM behavior change interventions.

Fifth, several other authors have analyzed BCTs used in interventions in their own field [37–39]. As in the SDM context, Instruction on how to perform a behavior was the most common BCT in effective management of physical activity for people with COPD interventions (69% of studies) [37] and in diabetic retinopathy attendance interventions (75% of studies) [39]. In other contexts, such as home-based cardiac rehabilitation, the most commonly used BCT is Social support [38]. The differences in most commonly used BCTs across all contexts can be explained by the nature of the determinants of the behavior (i.e., influential physical, psychological, automatic, or emotional factors) [13, 39].

Finally, this study meets its original objective, but is especially useful as a basis for a research agenda. We explored the functions and BCTs used in SDM implementation interventions. However, we did not explore the contextual factors, such as the influence of government policies (e.g., in favor of SDM or not), or the initiators of the studies (e.g., researchers or clinicians), factors that might have contributed to the success or failure of the interventions. The parent Cochrane review identified clinical conditions (e.g., cancer, cardiovascular diseases, psychiatric conditions), and health service environments in which the studies took place, but this study did not explore these factors [11]. We are thus not yet able to explain the mechanisms by which certain interventions (through the functions and BCTs used) did or did not produce their effect. Future studies could explore how diverse contexts could further influence the effects of interventions. Further research is also needed to determine if there is any further order distinguishable within the BCT and function combinations and to give more insight on the mechanisms by which they produce an effect, be it positive or not. 

This study has some limitations. We relied only on what was reported in the studies included in a Cochrane review. However, the Cochrane review relies upon robust methodology to identify all SDM implementation trials and so we were able to link functions and BCTs to the effectiveness or ineffectiveness of over 80 SDM implementation interventions. Second, our coding was dependent on the published information of the SDM implementation trials. These descriptions were often lacking in detail, and we did not contact the authors for more detail. The way authors reported their interventions (e.g., choice of verbs, verb tenses) greatly influenced our coding, so we may have under-coded or over-coded some interventions. For example, if an author reported that a participant was promised a reward before an intervention, the BCT could be coded as a type of incentive, but if they were reported only as having being rewarded afterwards, the BCT was coded as reward, and an unreported BCT incentive may have been missed. However, the findings presented here are the result of a consensus.

## Conclusions

We proposed a BCT taxonomy specific to the field of SDM implementation including some new BCTs that could be added to BCTTv1. This is the first attempt to examine the functions and BCTs of implementation interventions used in SDM implementation trials. Our analysis presents the most common functions and BCTs in SDM implementation interventions, both effective and ineffective, used singly or in combination. This could inform the choice of approaches and strategies for more effective SDM implementation interventions. Our new taxonomy could also improve the quality of SDM evidence by aiding in transparent reporting of interventions so that we can better measure the effects of their component parts. Further studies need to better investigate the interrelation between the chosen strategies (functions and BCTs), the implementation context and explanatory mechanisms.

## Supplementary information

**Additional file 1.** PRISMA checklist of information to include when reporting systematic reviews and meta-analyses.

**Additional file 2.** Examples of each function and behavior change technique used in the shared decision making implementation interventions.

**Additional file 3.** Behavior change techniques used in each shared decision making implementation intervention.

**Additional file 4.** Behavior change techniques and their effectiveness by population target.

## Data Availability

The datasets supporting the conclusions of this article are included within the article and its additional file.

## Abbreviations

BCT: Behavior change techniques
BCW: Behavior change wheel
DA: Patient decision aid
HP: Health professionals
SDM: Shared decision making
