# Supplementary material for: Towards a taxonomy of behavior change techniques for promoting shared decision making
Source: Implement Sci. 2020 Aug 20;15:67. doi: 10.1186/s13012-020-01015-w (PMC7439658; doi:10.1186/s13012-020-01015-w)
Supplement: Supplementary file 2 — Additional file 2. Examples of each function and behavior change technique used in the shared decision making implementation interventions. [file 13012_2020_1015_MOESM2_ESM.docx]

Additional file 2: Examples of each function and BCT used in the SDM implementation interventions

| **Function** | **Definition (1)** | **Quote illustrating the function** |
| --- | --- | --- |
| Education | Increasing knowledge or understanding. | Patients were invited to view a Web-based decision aid, a mailed paper version of the decision aid, or no pre-visit educational material. The Web-based decision aid (http://www.acorn.fap.vcu.edu/psa), developed by the authors, presented *information about prostate cancer, screening concepts, potential screening benefits, and known risks, as well as current uncertainties*… (Krist 2008) |
| Training | Imparting skills. | The second component was a *three-hour skill building workshop* provided by a trained facilitator external to the project… (Loh, 2007) |
| Enablement | Increasing means/reducing barriers to increase capability (beyond education and training) or opportunity (beyond environmental restructuring). | Three types of conversations that are crucial for talking about ACP in the nursing home were used for practising SDM… As a *homework assignment*, participants were to practise the ‘Option talk’ by engaging in conversations with residents about preferences in routine care situations, and thus to talk about the different care options. Conversations in crisis situations were used as a prototype for the ‘Decision talk’ in the second workshop (which took place after the homework assignment), since the urgency of crisis situations require that certain decisions have to be made… (Ampe 2017) |
| Modeling | Providing an example for people to aspire to or imitate. | Those clinicians delivering the decision aid watched an *8-min video demonstrating the use of the decision aid* and had an opportunity to ask questions.… (Warner 2015) |
| Persuasion | Using communication to induce positive or negative feelings or stimulate action. | *Physicians then calculated each patient’s absolute risk for stroke and myocardial infarction* on the basis of an adapted Framingham algorithm with the decision aid… *Individual prognosis was compared with age- and sex-adjusted population risk.* (Krones 2008) |
| Incentivization | Creating an expectation of reward. | They were also told that all participants *would receive a $25 stipend upon completion of the study*. (Dolan 2002) |
| Environmental restructuring | Changing the physical or social context. | In arm 3, clinicians received *a set of RA Choice cards* to be used during the visit. (Barton 2016) |
|  |  |  |
| **BCT** | **Definition** | **Quote illustrating the BCT** |
| Goal setting (behavior) | Set or agree on a goal defined in terms of the behavior to be achieved. | General health status, *care goals, and care plans were documented by the nurse and the patient together*… (Vestala 2013) |
| Problem solving | Analyze, or prompt the person to analyse, factors influencing the behavior and generate or select strategies that include overcoming barriers and/or increasing facilitators (includes ‘Relapse Prevention’ and ‘Coping Planning’). | Overcoming Barriers to Communication helps *patients think through various remaining barriers to communication and provides them with additional tools, such as the “I tool”* which teaches them to use statements with their providers that start with “I” such as “I want to know the results of my last lab test.” (Maclachlan 2016)  *Techniques of exploration, of coping with difficult situations*, and of inviting the patient into the decision-making process are practiced in role plays. (Bieber 2006) |
| Goal setting (outcome) | Set or agree on a goal defined in terms of a positive outcome of wanted behavior. | The fourth step enabled patients, in consultation with their pharmacist, *to define their treatment goals in terms of ideal HDL-C, LDL-C, blood pressure, CVD risk and cardiovascular age,* and to follow their progress over time… (Lalonde 2006) |
| Action planning | Prompt detailed planning of performance of the behavior (must include at least one of context, frequency, duration and intensity). Context may be environmental (physical or social) or internal (physical, emotional or cognitive) (includes ‘Implementation Intentions’). | The third step consisted of the *formulation of an action plan* for the next three months. (Lalonde 2006) |
| Review behavior goal(s) | Review behavior goal(s) jointly with the person and consider modifying goal(s) or behavior change strategy in light of achievement. This may lead to re-setting the same goal, a small change in that goal or setting a new goal instead of (or in addition to) the first, or no change. | The therapist had been trained to use this curve together with the patient to evaluate treatment progress. They had also learned to *discuss the SRS scores with the patients to establish what was working in the session and what could be improved*. (Rise 2012) |
| Commitment | Ask the person to affirm or reaffirm statements indicating commitment to change the behavior. | It concludes by asking participants which risk reducing option they plan to pursue, *reinforcing* *commitment to action*. (Sheridan 2014) |
| Monitoring of behavior by others without feedback | Observe or record behavior with the person’s knowledge as part of a behavior change strategy. | *Intervention sessions* *were audio recorded and reviewed by lead trainers and investigators* using a fidelity checklist. (Epstein 2017) |
| Feedback on behavior | Monitor and provide informative or evaluative feedback on performance of the behavior (e.g. form, frequency, duration, intensity). | For each participant, two audio-taped consultations were transcribed and analysed by the research team using the Decision Analysis System for Oncology. *Confidential written feedback concerning these tapes was provided at the end of the workshop*. (Bernhard 2011) |
| Self-monitoring of behavior | Establish a method for the person to monitor and record their behavior(s) as part of a behavior change strategy. | The features of MyAsthma (Supplemental Appendix 1) include identification of parents’ concerns and goals for asthma treatment; *monthly tracking of symptoms, medication side effects*, and progress toward goals; asthma educational content including videos; and access to the child’s asthma care plan. (Fiks 2015) |
| Monitoring outcome(s) of behavior without feedback | Observe or record outcomes of behavior with the person’s knowledge as part of a behavior change strategy. | The fourth step enabled patients, in consultation with their pharmacist, to define their treatment goals in terms of ideal HDL-C, LDL-C, blood pressure, CVD risk and cardiovascular age, *and to follow their progress over time*… (Lalonde 2006) |
| Biofeedback | Provide feedback about the body (e.g. physiological or biochemical state) using an external monitoring device as part of a behavior change strategy. | For patients randomized to the decision aid, a study coordinator collected each of the variables needed to populate the quantitative probability web tool, asked the treating clinician to sign off on their accuracy, and *calculated the patient’s pretest probability of acute coronary syndrome,* incorporating the result of the first troponin test but prior to subsequent biomarker testing… The treating clinician, after evaluating the patient and the results of the initial ECG and cardiac troponin tests, then used the decision aid to educate the patient about the results of the two tests, the potential need for observation and further cardiac testing, subsequent cardiac troponin testing to definitively rule out acute myocardial infarction, if required, and their *personalized 45 day risk for acute coronary syndrome*… (Hess 2016) |
| Feedback on outcome(s) of behavior | Monitor and provide feedback on the outcome of performance of the behavior. | The intervention thus consisted of systematically *using the ORS and SRS scales to assess feedback from the patient on treatment outcome* and the quality of the session. (Rise 2012) |
| Social support (unspecified) | Advise on, arrange or provide social  support (e.g. from friends, relatives, colleagues,’ buddies’ or staff) or noncontingent praise or reward for performance of the behavior. It includes  encouragement and counseling, but only when it is directed at the behavior. | Training included the rationale for the use of patient feedback and feedback scales, the practical use of the feedback scales, and how to incorporate the use of data from the scales in the treatment process + *counseling*. (Rise 2012) |
| Social support (practical) | Advise on, arrange, or provide practical help (e.g. from friends, relatives, colleagues, ‘buddies’ or staff) for performance of the behavior. | The physician’s role was *to clarify any questions regarding the patient’s medical profile pertinent to the consideration of HRT* and to discuss the patient’s current or future intentions regarding HRT. (Deschamps 2004) |
| Social support (emotional) | Advise on, arrange, or provide emotional social support (e.g. from friends, relatives, colleagues, ‘buddies’ or staff) for performance of the behavior. | These men also were encouraged to participate in deciding which treatment option was best for them, and *to bring their spouse/significant other(s) to the treatment consultation.* (Davison 1997) |
| Instruction on how to perform the behavior | Advise or agree on how to perform the behavior (includes ‘Skills training’). | The doctors and/or the nurses who were primarily involved in the diabetes care of the practice *attended a short training session lasting between 1 and 2 h on how to use the PANDAs decision aid*. (Mathers 2012) |
| Information about health consequences | Provide information (e.g. written, verbal, visual) about health consequences of performing the behavior. | Briefly, the tool is designed to enable clinicians to discuss with patients the *potential advantages and disadvantages of adding an agent from 1 of the following antihyperglycemic classes to their regimen*: metformin, insulin, thiazolidinediones, exenatide, and sulfonylureas… The tool consists of 6 cards that describe the possible effects of the medications on 6 outcomes: “Weight Change,” “Low Blood Sugar (Hypoglycemia),” “Blood Sugar (A1c Reduction),” “Daily Routine,” “Daily Sugar Testing (Monitoring),” and “Side Effects”. (Mullan 2009) |
| Salience of consequences | Use methods specifically designed to emphasize the consequences of performing the behavior with the aim of making them more memorable (goes beyond informing about consequences). | Possible treatment options for breast reconstruction with their indications/contraindications, advantages/disadvantages, expected postoperative course, aesthetic results with *pre- and postoperative photos for each type of reconstruction and the most common complications* and their probabilities. (Causarano 2015) |
| Information about social and environmental consequences | Provide information (e.g. written, verbal, visual) about social and environmental consequences of performing the behavior. | In addition to usual primary care, intervention patients received a decision aid (a tailored pictographic 10-year fracture risk estimate, absolute risk reduction with bisphosphonates, side effects, and *out-of-pocket cost*), and control patients received a standard brochure. (Montori 2011) |
| Information about emotional consequences | Provide information (e.g. written, verbal, visual) about emotional consequences of performing the behavior. | The decision aid also informed about the possibility of breast reconstruction for these women. Moreover, the decision aid shortly stated the issue of leaving the lump for a few additional months in the breast and that *in consequence* *for some women immediate surgery might be psychologically more beneficial*. (Vodermaier 2009) |
| Demonstration of the behavior | Provide an observable sample of the performance of the behavior, directly in person or indirectly e.g. via film, pictures, for the person to aspire to or imitate (includes ‘Modeling’). | Participating clinicians were oriented during a 1-hour training session given by the lead investigator (E.P.H.) as well as *a brief (3 min) demonstration from the study coordinator on how to use the decision aid* before meeting the first enrolled patient and as needed. (Hess 2012) |
| Information about others’ approval | Provide information about what other people think about the behavior. The  information clarifies whether others will like, approve or disapprove of what the person is doing or will do. | In response to focus groups with stakeholders, *these materials incorporated images of African Americans and personalized viewpoints on safety and effectiveness of anti-depressant medication* and counseling, spirituality, and suicide prevention from actual African American patients, clinicians, community members, and clergy. (Cooper 2013) |
| Prompts/cues | Introduce or define environmental or social stimulus with the purpose of prompting or cueing the behavior. The prompt or cue would normally occur at the time or place of performance. | To optimize implementation, the checklist was bundled with a 1-hour interactive training, a brief refresher training, tools to monitor implementation, and *laminated checklists for use as prompts*, constituting the FCR checklist intervention…‍ (Cox 2017) |
| Behavioral practice/rehearsal | Prompt practice or rehearsal of the performance of the behavior one or more times in a context or at a time when the performance may not be necessary, in order to increase habit and skill. | The training consisted of a 7-h interactive face-to-face workshop with one to two follow-up telephone calls over 2 months. The elements of this training were evidence-based, incorporating presentation of principles, a video modelling ideal behavior and *role-play practice*. (Bernhard 2011) |
| Generalization of target behavior | Advise to perform the wanted behavior, which is already performed in a particular situation, in another situation. | In between workshop 1 and workshop 2 (3–4 weeks), *all participants are assigned homework concerning informal conversations about end-of-life issues*. (Ampe 2017) |
| Credible source | Present verbal or visual communication from a credible source in favour of or against the behavior. | In each wave, at each site, BRIDGES was taught once a week for eight consecutive weeks, and each class was 2.5 h long. *Classes were taught by a team of two trained instructors who publicly acknowledge that they are in recovery from a mental illness*. (Pickett 2012) |
| Pros and cons | Advise the person to identify and compare reasons for wanting (pros) and not wanting to (cons) change the behavior (includes ‘Decisional balance’). | In the decision aid group, participants were taken through a presentation of the individualised benefits and potential harms of warfarin treatment and *were invited to weigh up the advantages and disadvantages of treatment* before coming to a shared decision with the clinic doctor. (Thomson 2007) |
| Material incentive (behavior) | Inform that money, vouchers or other valued objects will be delivered if and only if there has been effort and/or progress in performing the behavior (includes ‘Positive reinforcement’) | They were also told that all participants *would receive a $25 stipend upon completion of the study*. (Dolan 2002) |
| Material reward (behavior) | Arrange for the delivery of money, vouchers or other valued objects if and only if there has been effort and/or progress in performing the behavior (includes ‘Positive reinforcement’). | Pharmacists *received a total of Canadian $45 per patient recruited in partial compensation* for their time. (Stacey 2006) |
| Pharmacological support | Provide, or encourage the use of or adherence to, drugs to facilitate behavior change. | Patients and clinicians were to review the decision aid, *deliberate about whether to start bisphosphonates, and make a decision together at that time or at a later time*. (Leblanc 2015b) |
| Reduce negative emotions | Advise on ways of reducing negative emotions to facilitate performance of the behavior (includes ‘Stress Management’). | The programme was developed by the Dresden group (SiK, TZ) based on a previously developed programme. The cognitive behavioral programme *focuses on experience with management of stress and anxieties* and aims to enhance participants’ resources as well as stress coping strategies. (Köpke 2014) |
| Adding objects to the environment | Add objects to the environment in order to facilitate performance of the behavior. | Each individual *was given a blank audio tape*, and was responsible for asking the physician to tape their consultation. (Davison 1997) |

Consult the full taxonomy (BCTTv1) for more details on the definitions of BCTs (1).

Quotes are for information purposes only. Consult the entire article as needed to better understand the context (2).

Italicized portions of the sentence help to better identify the part of the sentence that refers to the function or the BCT.

References have been removed from the quotes for the sake of presentation.

**References**

1. Michie S, Richardson M, Johnston M, Abraham C, Francis J, Hardeman W, et al. The behavior change technique taxonomy (v1) of 93 hierarchically clustered techniques: building an international consensus for the reporting of behavior change interventions. Annals of behavioral medicine. 2013;46(1):81-95.

2. Légaré F, Adekpedjou R, Stacey D, Turcotte S, Kryworuchko J, Graham ID, et al. Interventions for increasing the use of shared decision making by healthcare professionals. 2018(7).
