# Supplementary material for: Towards a taxonomy of behavior change techniques for promoting shared decision making
Source: Implement Sci. 2020 Aug 20;15:67. doi: 10.1186/s13012-020-01015-w (PMC7439658; doi:10.1186/s13012-020-01015-w)
Supplement: Supplementary file 4 — Additional file 4. Behavior change techniques and their effectiveness by population target. [file 13012_2020_1015_MOESM4_ESM.docx]

Additional file 4: Behavior change techniques and their effectiveness by population target

| **BCTs** | **Effect** | **Target population (N = 87)** | | | |
| --- | --- | --- | --- | --- | --- |
|  |  | N = 87 | HP (n = 15) | Patients (n = 44) | Both (n = 28) |
| - 1. *Goal setting (behavior)* | | *8* | - | 4 | 4 |
|  | *Yes* | *2* | - | - | 2 |
|  | *No* | *6* | - | 4 | 2 |
| - 1. *Problem solving* | | *9* | 2 | 2 | 5 |
|  | *Yes* | *3* | - | - | 3 |
|  | *No* | *6* | 2 | 2 | 2 |
| - 1. *Goal setting (outcome)* | | *1* | - | 1 | - |
|  | *Yes* | *-* | - | - | - |
|  | *No* | *1* | - | 1 | - |
| - 1. *Action planning* | | *3* | - | 1 | 2 |
|  | *Yes* | *1* | - | - | 1 |
|  | *No* | *2* | - | 1 | 1 |
| - 1. *Review behavior goal(s)* | | *2* | - | - | 2 |
|  | *Yes* | *-* | - | - | - |
|  | *No* | *2* | - | - | 2 |
| *1.9. Commitment* | | *1* | - | 1 | - |
|  | *Yes* | *-* | - | - | - |
|  | *No* | *1* | - | 1 | - |
| *2.1. Monitoring of behavior by others without feedback* | | *1* | - | - | 1 |
|  | *Yes* | *-* | - | - | - |
|  | *No* | *1* | - | - | 1 |
| **2.2. Feedback on behavior** | | **13** | 8 | - | 5 |
|  | **Yes** | **8** | 5 | - | 3 |
|  | **No** | **5** | 3 | - | 2 |
| *2.3. Self-monitoring of*  *behavior* | | *3* | 1 | - | 2 |
|  | *Yes* | *1* | - | - | 1 |
|  | *No* | *2* | 1 | - | 1 |
| *2.5. Monitoring of outcome(s) of behavior without feedback* | | *1* | - | 1 | - |
|  | *Yes* | *-* | - | - | - |
|  | *No* | *1* | - | 1 | - |
| **2.6. Biofeedback** | | **2** | - | 1 | 1 |
|  | **Yes** | **1** | - | - | - |
|  | **No** | **-** | - | 1 | 1 |
| *2.7. Feedback on outcome(s) of behavior* | | *1* | - | - | 1 |
|  | *Yes* | *-* | - | - | - |
|  | *No* | *1* | - | - | 1 |
| *3.1. Social support (unspecified)* | | *28* | 2 | 15 | 11 |
|  | *Yes* | *8* | 1 | 3 | 4 |
|  | *No* | *19* | 1 | 12 | 6 |
|  | *Error* | *1* | - | - | 1 |
| *3.2. Social support (practical)* | | *26* | 4 | 9 | 13 |
|  | *Yes* | *12* | 3 | 2 | 7 |
|  | *No* | *13* | 1 | 7 | 5 |
|  | *Error* | *1* | - | - | 1 |
| *3.3. Social support (emotional)* | | *4* | - | 4 | - |
|  | *Yes* | *1* | - | 1 | - |
|  | *No* | *3* | - | 3 | - |
| **4.1. Instruction on how to**  **perform the behavior** | | **43** | 12 | 8 | 23 |
|  | **Yes** | **22** | 6 | 1 | 15 |
|  | **No** | **20** | 6 | 7 | 7 |
|  | Error | 1 | - | - | 1 |
| *5.1. Information about health consequences* | | *44* | 2 | 27 | 16 |
|  | *Yes* | *21* | 1 | 9 | 11 |
|  | *No* | *23* | - | 18 | 5 |
| *5.2. Salience of consequences* | | *3* | - | 3 | - |
|  | *Yes* | *-* | - | - | - |
|  | *No* | *3* | - | 3 | - |
| 5.3. Information about social and environmental consequences | | 6 | - | 5 | 1 |
|  | Yes | 3 | - | 2 | 1 |
|  | No | 3 | - | 3 | - |
| *5.6. Information about emotional consequences* | | *2* | - | 1 | 1 |
|  | *Yes* | *-* | - | 1 | 1 |
|  | *No* | *2* | - | - | - |
| **6.1. Demonstration of the**  **behavior** | | **19** | 6 | - | 13 |
|  | **Yes** | **12** | 3 | - | 9 |
|  | **No** | **6** | 3 | - | 3 |
|  | Error | 1 | - | - | 1 |
| *6.3. Information about others’ approval* | | *1* | - | - | 1 |
|  | *Yes* | *-* | - | - | - |
|  | *No* | *1* | - | - | 1 |
| *7.1. Prompts/cues* | | *7* | 3 | 1 | 3 |
|  | *Yes* | *2* | - | - | 2 |
|  | *No* | *5* | 3 | 1 | 1 |
| 8.1. Behavioral  practice/rehearsal | | 23 | 11 | 4 | 8 |
|  | Yes | 11 | 5 | - | 6 |
|  | No | 11 | 6 | 4 | 1 |
|  | Error | 1 | - | - | 1 |
| *8.6. Generalization of target behavior* | | *2* | 2 | - | - |
|  | *Yes* | *-* | - | - | - |
|  | *No* | *2* | 2 | - | - |
| *9.1. Credible source* | | *23* | 2 | 12 | 9 |
|  | *Yes* | *9* | 2 | 3 | 4 |
|  | *No* | *13* | - | 9 | 4 |
|  | *Error* | *1* | - | - | 1 |
| *9.2. Pros and cons* | | *5* | - | 4 | 1 |
|  | *Yes* | *1* | - | 1 | - |
|  | *No* | *4* | - | 3 | 1 |
| *10.1. Material incentive (behavior)* | | *7* | - | 3 | 4 |
|  | *Yes* | *3* | - | 1 | 2 |
|  | *No* | *4* | - | 2 | 2 |
| *10.2. Material reward (behavior)* | | *11* | 1 | 4 | 6 |
|  | *Yes* | *3* | 1 | - | 2 |
|  | *No* | *7* | - | 4 | 3 |
|  | Error | 1 | - | - | 1 |
| **11.1. Pharmacological support** | | **6** | - | 3 | 3 |
|  | **Yes** | **5** | - | 2 | 3 |
|  | **No** | **1** | - | 1 | - |
| *11.2. Reduce negative emotions* | | *1* | - | 1 | - |
|  | *Yes* | *-* | - | - | - |
|  | *No* | *1* | - | 1 | - |
| *12.5. Adding objects to the*  *environment* | | *10* | 4 | 1 | 5 |
|  | *Yes* | *4* | 1 | 1 | 2 |
|  | *No* | *6* | 3 | - | 3 |

HP: health profesional;

In bold, BCTs associated with more positive effect studies than no-effect studies.

In italics, BCTs associated with more no-effect studies than positive studies.

In Roman type, the BCTs associated with as many positive effect studies as no-effect studies.
